# Sexual Behavior and Condom Use among Gay Men, Female Sex Workers, and Their Customers: Evidence from South Korea

**DOI:** 10.1371/journal.pone.0066867

**Published:** 2013-06-24

**Authors:** Minsoo Jung

**Affiliations:** 1 Department of Medical Oncology, Dana-Farber Cancer Institute, Boston, Massachusetts, United States of America; 2 Department of Health Science, Dongduk Women’s University, Seoul, South Korea; University of Illinois at Chicago, United States of America

## Abstract

**Background:**

Despite the significance for sexually transmitted diseases (STD) control in East Asia, few studies have examined the relationship between high-risk sexual behavior and condom use. We investigated how three sexually vulnerable groups for STDs show differences in condom use behaviors (CUBs) depending on their STD infection.

**Methods:**

The source of data came from the National Survey for STD Prevalence Rate and Sexual Behavior of the High-Risk Sexual Community. The effects of behavioral determinants on CUBs were estimated by using path analysis models. An 11-item questionnaire assessing subjects’ health risk behaviors, sexual beliefs, sexual risk behaviors, and condom use.

**Results:**

Condom use was higher for men who have sex with men (MSM; *n* = 108) when they were bisexuals and had high self-efficacy, for Johns (Johns; *n* = 118) when they had experience of STD infection, and for female sex workers (FSWs; *n* = 1,083) when they had high self-efficacy, did not engage in drunken sex, and were anxious about infection. Regardless of whether they were infected with STDs, FSWs always used condom when they had high sexual beliefs. On the contrary, Johns exhibited a negative relationship between sexual risk behavior and condom use when they had experience of STD infection. The variable commonly significant to all three groups was the number of sex partners; but it exhibited a positive relationship with MSMs and Johns, and a negative one with FSWs.

**Conclusions:**

CUBs were related to sexual beliefs as well as sexual risk behavior. At the same time, the experience of STD infection mediated the relationship between the two. Therefore, we need to draw social attention to promote safer sex among STD-vulnerable groups.

## Introduction

Sexually transmitted diseases (STDs) have attracted attention as a major public health issue, and their management remains a daunting challenge [1]. Because of the difficulty in accessing groups with higher prevalence rates of STDs, sufficient information about their sexual risk behaviors is lacking, which has boosted the prevalence rate of STDs [2], [3]. However, preventive intervention in the unseen communities is important because such groups are insensitive to condom use despite concurrent sexual partnerships (CSPs) [4], [5]. For example, groups such as men who have sex with men (MSMs), female sex workers (FSWs), immigrant workers [5]–[7], and male customers who solicit sex from prostitutes (Johns) have recently come under surveillance by the general population [8]. Despite the significance of these groups for STD control in East Asia, few studies have investigated their condom use in terms of the social context.

Attitudes toward behavior, subjective norms, and perceived behavioral control constitute crucial elements of behavioral decision making according to the theory of planned behavior (TPB), a classical theory for the explanation and prediction of condom use [9]. The merit of TPB is its ability to explain human behaviors as a combination of norms, attitudes, and self-efficacy. However, this is based on individual rationality, so that its applicability becomes limited in the case of behaviors such as condom use that requires relational/coordinative decision making and is influenced by social pressure [3]. By contrast, the health belief model (HBM) illustrates human behaviors with cognitive elements such as the perceived threat of illness, outcome expectation, and perceived self-efficacy [10]. It also provides a crucial platform for predicting condom use behaviors (CUBs) [11]. As both theories cover general populations such as university students [11], [12], a separate tool is required to explain the CUB of a high-risk sexual community in an entirely different sexual context.

Risky sexual behaviors appear in a couple of groups that have differentiated characteristics from the normal population [6]. The prevalence of STDs tends to be higher in urban residents, unmarried individuals, and young adults [13]. One group often neglected in these classifications, however, is FSWs [14]–[16]. Although the sex industry today is very extensive and diversified, FSWs remain generally socially excluded and vulnerable, particularly in terms of their health. What is especially problematic is that although FSWs can easily be infected by STDs and can spread them to many people, they are not being properly managed as a vulnerable population in South Korea. Changes in the extent and status of sexual diseases are collected through a sentinel surveillance system and various regulations and sexual disease examination systems have been established. However, because sex labor itself is not socially approved, the position of FSWs is uncertain and precarious. This has rapidly forced the sex industry to operate underground and has made the recruitment of FSWs all the more difficult. Therefore, it is necessary to examine the CUB of high-risk sexual groups.

Numerous studies have investigated typical high-risk groups such as FSWs and MSMs [17]–[20]. In this process, a number of variables of condom use have been reviewed [21], but the review of the causal and mediating mechanism for those variables remains relatively unsophisticated. In particular, these studies neglected to consider the social characteristics of sexual risk behaviors and sexual beliefs that are featured in the above groups. Moreover, few studies have examined the relationship between the experience of STDs and condom use. After generating primary data on three sexually vulnerable groups for STDs, we investigated how the three groups show differences in CUBs, along with their sexual risk factors. At the same time, we examined the causal relationship among the sexual determinants on CUBs, thereby comparing the groups with and without the experience of STDs in terms of the mediating mechanism for condom use. The study thereby aims to ensure a more refined and systematic prevention of STDs and policy support for a safe-sex practice program for high-risk sexual behavior groups.

## Materials and Methods

### Ethical Issues

This research passed the institutional review board of Seoul National University Hospital in May 14th, 2008. In order to protect vulnerable research subjects, every actual site investigation was performed by receiving written informed consent from the participants. During the investigation process, absolutely no information that could distinguish individual respondents was collected, and clinical specimens were classified using bar codes. Investigation results were made so that only the individual himself was able to confirm through the automatic response system of the Science Research Center in Seoul, and the secrecy of all information was fully assured.

### Study Sample

A Korean government-sanctioned survey was conducted from June to November 2008. This was the first investigation on the STD prevalence rate and sexual behavior of the high-risk sexual community. For the field investigation, each dispatched team consisted of one physician, two clinical pathologists, two masters of public health, and one coordinator. Trained interviewers facilitated FSWs to answer a questionnaire on sexual behaviors, while laboratory technicians gathered urine, oropharyngeal swabs, and blood samples. The following biological samples were tested: treponema pallidum antibodies (TP-PA), which causes syphilis, and urine polymeras chain reaction (PCR) for chlamydia trachomatis and neisseria gonorrhea. A pathologic test for clinical specimens was carried out by the Seoul Medical Science Institute.

The prostitution occurring in established locations is largely divided into two types. Establishments with full-time sex workers mainly deal in sex trafficking and are densely populated in red-light districts (RLDs). In these areas, FSWs maintain their livelihood exclusively through prostitution. In contrast, establishments that offer prostitution as a side-business mainly sell alcohol or massage services, and try to supplement these by also offering prostitution. The FSWs in the former type of prostitution establishment exhibit a typical and consistent sexual behavior that differs from FSWs directly or indirectly making contact with clients and forming relationships, but not at a set location. Brothels are generally located at the periphery of busy downtown areas with a high volume of population movement. An RLD usually has about 10–50 brothels located in close proximity to each other, and they engage in solicitation of clients as a group. Each brothel usually has about 2–10 FSWs who live with their pimps and stay there for a long time. Accordingly, RLDs in Korea have the most appropriate conditions for FSW recruitment using time location sampling (TLS). TLS is based on the tendency of ‘daily unnoticed community,’ [22] and is useful for groups such as MSMs or FSWs that can only be approached in a particular location [23], [24].

For this study, the 42 national RLDs (with approximately 6,000 FSWs) listed by the Ministry of Gender Equality and Family were stratified according to city population and brothel size, and 13 areas were selected. With regard to the sampling location for the recruitment of MSMs and FSWs, we used the national center for HIV/AIDS of the three largest areas nationwide (*n* = 108) and the RLDs of 13 areas (*n* = 1,083), respectively. However, since Johns are not an exclusive community and has no social network or such inclinations, the John School, the only location where they are guaranteed to assemble, was used as the sampling frame. We conducted convenience sampling of eight John Schools, the only spot where Johns are accessible (*n* = 118). The John School in South Korea is a type of probation facility which aims to prevent the recurrence of sex trafficking through an eight-hour sex education session for male clients of prostitutes. The response rates are reported as 92% for FSWs, 81% for MSMs, and 83% for Johns, respectively.

### Study Variables

**Figure 1:**
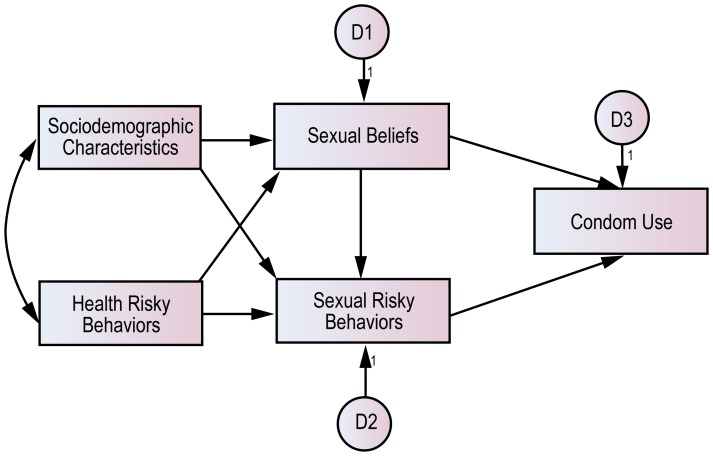
The hypothetical model of this study.

The sexual risk behavior measurements of this study set the groundwork for behavioral surveillance surveys whose validity was verified through an internationally recognized process and its Korean version [25], [26]. We focused on anxiety about STDs as an attitude factor regarding self-efficacy [12], [27] and condom use, which are stressed by both the TPB and the HBM, and simplified anxiety about STDs as the factor of sexual beliefs. However, sexual risk behavior, which reflected these groups’ unique characteristics more than did individual perception factors, was developed as a separate factor. In addition, according to their individual characteristics, the factors of health risk behavior and socio-demographic characteristics were measured simultaneously (Figure 1). For analysis, we developed structural path models for the three groups.

### Independent Variables

From the exploratory factor analyses, the following four factors were defined as independent variables for the prediction of CUBs. Each factor referred to a single factor with an eigen value of 1 or more in the principal component analyses with oblique rotation, thereby showing an outcome consistent with the model designed herein. The reliability for each question regarding the sexual risk behaviors measured by three groups was determined for each dimension, using Cronbach’s alpha coefficient.

Sexual Belief Factors (Factor loading = 57.2; Cronbach’s α = 0.74): These factors include the degree of anxiety over STD infections and self-efficacy. Anxiety over STD infections was measured by subjective feelings about the risk of STD infections [8], while self-efficacy was measured by the questions about how easy or difficult it would be to find condoms that fit properly, to put a condom on correctly, and to use a condom throughout the sexual encounter [28]. Responses varied from 1 (very low) to 4 (very high) for both variables.

Sexual Risk Factors (Factor loading = 52.0; Cronbach’s α = 0.70): These factors include age at first sexual experience and the number of sex partners. The latter variable was gauged by the average number of daily sex partners for the previous month. In South Korea, the ratio of bisexuals was reported to be high among MSMs [3], so the experience of having had a bisexual relationship was additionally measured in the case of MSMs.

Health Risk Factors (Factor loading = 50.0; Cronbach’s α = 0.87): These factors comprise the habits of smoking and drinking alcohol. Smoking was measured via a 5-point scale based on the frequency of smoking, with response options collapsed into categories including: Two packs or more; One pack; Half pack; Half pack or less; and Does not smoke. The factor of drinking alcohol was gauged via a 3-point scale, which was collapsed into the following categories: Often; Usually; and Never. Given the close inverse correlation between drinking and condom use [29], we examined whether sexual experiences usually occur when the participants are inebriated.

Sociodemographic Factors (Factor loading = 33.5; Cronbach’s α = 0.75): These factors include education level and the number of social relationships. Education level was measured via a 4-point scale, which were collapsed into the following categories: Elementary school or less; Middle school; High school; and College or post-graduate. Social relationships included usual phone conversations, direct encounters, and conversations. Having strong social relationships can become a manifestation of having solid social support.

### Mediating Variable

As a mediating variable influential on the use of condoms, whether the person is or was ever infected with as STD was also considered. A current infection was confirmed through a specimen test, and a previous infection was identified through a survey, which was limited to cases diagnosed by doctors. An infection case was coded as 1, and a non-infection one as 0. Thus this mediating variable was used as a dummy variable in the regression model, and as a categorical factor in the path analysis to understand the mediating effects of the STD infection.

### Dependent Variable

Respondents were asked about their condom use practice in sexual relations in the previous month, with response options collapsed into categories including: Almost; Frequently; Often; Never; Do not know; and Refused to answer. Here, sex partners were casual partners. To prevent the problem of reverse causality between a mediating variable and a dependent variable, we measured the frequency of condom use for the current month and the experience of STD infection in terms of lifetime infection.

### Statistical Analyses

The following analyses were conducted. 1) Calculation of the frequency and percentage of the general characteristics of the samples. 2) Calculation of the STD prevalence rates of the objects of the investigation. 3) Ordinary least squares (OLS) regression analyses, where factors related to condom use in STD-vulnerable groups were analyzed and compared. However, to satisfy independent and identical distribution, a condition of regression analysis, the number of sex partners and social support variables were applied to the model after undergoing log transformation. 4) Path analyses were conducted on groups with and without STD infection experience. All missing values were excluded using a pairwise method. Statistical analyses were performed using STATA ver. 10.0 (STATA, College Station, TX) and AMOS ver. 7.0 (IBM SPSS Institute, Chicago, IL).

## Results

### Sample Description

The dominant age group in MSMs and FSWs was people in their 20s: 42.6% and 66.8%, respectively. However, those in their 30s were the largest group (43.2%) in Johns (Table 1). When asked if they were inebriated during sexual intercourse, 78.0% Johns responded “often” or “usually”, while 74.1% in FSWs replied “never.” On average FSWs had their first sexual experience at a relatively early age; 82.1% experienced it as minors (under 20). In consideration of the nature of their employment, FSWs had the largest number of sex partners; 66.3% had more than 10 per week on average. 50.9% in MSMs had had bisexual intercourse during the previous year. The FSWs were concerned about STDs infection; 37.7% recorded that they were somewhat anxious. Majorities in all three groups had medium-high self-efficacy. A high 60.2% in MSMs and 60.0% in FSWs were or had been infected with STDs. Lastly, the FSWs used condoms most frequently, and Johns used them least. Particularly, 28.0% of Johns said that they never used condoms.

**Table 1:** General Sample Characteristics.

		MSMs (n = 108)		Johns (n = 118)		FSWs (n = 1,083)	
		n	%	n	%	n	%
Age	<20	2	1.9	0	0.0	5	0.5
(yrs)	20–29	46	42.6	41	34.7	723	66.8
	30–39	43	39.8	51	43.2	288	26.6
	≥40	16	14.8	26	22.0	56	5.2
	Missing	1	0.9	0	0.0	11	1.0
Education	Elementary school or less	0	0.0	0	0.0	18	1.7
	Middle school	0	0.0	5	4.2	173	16.0
	High school	24	22.2	44	37.3	782	72.2
	College or post-graduate	83	76.9	69	58.5	95	8.8
	Missing	1	0.9	0	0.0	15	1.4
Number of	None	3	2.8	0	0.0	69	6.4
social	1∼5	23	21.3	25	21.2	262	24.2
relationships	6∼10	23	21.3	23	19.5	292	27.0
(person)	11∼20	27	25.0	20	16.9	281	25.9
	21∼30	17	15.7	20	16.9	101	9.3
	>30	13	12.0	20	16.9	78	7.2
	Missing	2	1.9	10	8.5	0	0.0
Smoking	Two packs or more	2	1.9	7	5.9	251	23.2
(per day)	One pack	25	23.1	58	49.2	581	53.6
	Half pack	14	13.0	16	13.6	128	11.8
	Half pack or less	4	3.7	1	0.8	31	2.9
	Don’t smoke	62	57.4	36	30.5	85	7.8
	Missing	1	0.9	0	0.0	7	0.6
Drunken sex	Often	9	8.3	27	22.9	39	3.6
	Usually	47	43.5	65	55.1	223	20.6
	Never	51	47.2	26	22.0	803	74.1
	Missing	1	0.9	0	0.0	18	1.7
Age at first sex	<14	10	9.3	0	0.0	11	1.0
(yrs)	14∼17	18	16.7	22	18.6	357	33.0
	18∼20	21	19.4	45	38.1	521	48.1
	>20	57	52.8	50	42.5	143	13.2
	Missing	2	1.8	1	0.8	51	4.7
Number of	0∼3	47	43.5	65	55.1	19	1.8
sex partners	4∼6	21	19.4	35	29.7	55	5.1
(person)	7∼10	11	10.2	12	10.2	170	15.7
	>10	17	15.7	6	5.1	718	66.3
	Missing	12	11.1	0	0.0	121	11.2
Bisexual	Never	49	45.4	NA		NA	
relationships	Once or twice	44	40.7				
	Frequently	11	10.2				
	Missing	4	3.7				
Anxiety for	Very high	8	7.4	3	2.5	87	8.0
STDs infection	High	34	31.5	28	23.7	408	37.7
	Low	40	37.0	69	58.5	339	31.3
	Very low	24	22.2	18	15.3	232	21.4
	Missing	2	1.9	0	0.0	17	1.6
Self-efficacy	Very high	32	29.6	57	48.3	227	21.0
	High	44	40.7	53	45.0	528	48.8
	Low	19	17.6	7	5.9	291	26.9
	Very low	8	7.4	0	0.0	17	1.6
	Missing	5	4.6	1	0.8	20	1.8
STDs infection	Never	65	60.2	61	51.7	650	60.0
	Ever	43	39.8	57	48.3	432	39.9
	Missing	0	0.0	0	0.0	1	0.1
Condom use	Almost	52	48.1	57	48.3	564	52.1
	Frequently	21	19.4	7	5.9	273	25.2
	Often	14	13.0	20	17.0	209	19.3
	Never	1	0.9	33	28.0	10	0.9
	Missing	20	18.5	1	0.8	27	2.5

### STDs Prevalence

The prevalence rate of the three groups was as follows (Table 2). MSMs had the highest rates at 22 (20.4%) TP-PA-positive cases and 7 HIV-infected participants. Johns had 6 (5.8%) chlamydia-positive cases. FSWs had 130 (12.5%) chlamydia-positive and 104 (9.8%) TP-PA -positive cases.

**Table 2:** Prevalence of Sexually Transmitted Diseases (STDs) among MSMs, Johns, and FSWs.

Type	MSMs (N = 108)		Johns (N = 118)		FSWs (N = 1,083)	
	N	PositiveN, (%)	N	PositiveN, (%)	N	PositiveN, (%)
TP-PA	108	22 (20.4)	NA	–	1,065	104 (9.8)
N. gonorrhea	106	0 (0)	117	0 (0)	1,060	27 (2.6)
C. trachomatis	106	2 (1.9)	117	6 (5.8)	1,060	130 (12.5)
HIV	108	7 (6.5)	NA	–	1,060	0 (0.0)

### Multivariate Analyses of Condom Use by Sexual Risk Behavior and STDs Infection

Multivariate regression analyses were conducted of five factors in the CUBs of the three STD vulnerable groups, with the following findings (Table 3). In MSMs, condom use was more frequent in individuals of an older age (*B* = 0.030, *p*<0.01), at an older age at first sexual encounter (*B* = −0.092, *p*<0.001), with fewer sex partners (*B* = −0.027, *p*<0.01), with more experience of heterosexual relationships (*B* = 0.030, *p*<0.05), and with higher self-efficacy (*B* = 0.330, *p*<0.05). In other words, condom use tended to prevail among those with less sexual risk behaviors and stronger sexual belief.

**Table 3:** Multivariate Analyses of Condom Use by Sexual Risk Behavior and STDs Infection among MSMs, Johns, and FSWs.

Factor		MSMs (N = 108)		Johns (N = 118)		FSWs (N = 1,083)	
	Variable	*B*	S.E.	*B*	S.E.	*B*	S.E.
Social-demographic	Age	0.030^**^	0.012	0.000	0.013	−0.009^*^	0.004
Characteristics	Educational Level	0.137	0.297	0.306	0.226	0.066	0.045
	Social Support ^ln^	**−**0.099	0.105	**−**0.004	0.202	**0.002** †	0.001
Health Risk Behavior	Smoking	**−**0.234	0.213	0.204	0.298	**−0.041** †	0.023
	Drunken Sex	**−**0.352	0.216	0.110	0.314	−0.153^***^	0.047
Sexual Risk Behavior	Age at First Sex	−0.092^***^	0.019	0.000	0.052	0.009	0.010
	# of Sex Partners ^ln^	−0.027^**^	0.010	−0.079^*^	0.035	0.005^**^	0.002
	Bisexuality	0.379^*^	0.182				
Sexual Belief	Anxiety for STDs	0.055	0.121	0.048	0.182	0.100^***^	0.028
	Self-efficacy	0.330^*^	0.128	0.120	0.160	0.410^***^	0.034
STDs Infection	Ever infected	0.073	0.223	**0.504** †	0.265	−0.133^**^	0.050
Fit	N	108		118		1056	
Statistics	F-ratio	4.280		1.218		28.398	
	p-value	p<0.001		n.s.		p<0.001	
	adjusted R^2^	0.353		0.103		0.214	

*Note*: *B*: standardized beta; S.E.: standard error; ^ln^: log transformed.

† p<0.10; ^*^p<0.05; ^**^p<0.01; ^***^p<0.001.

In Johns, condom use was higher in individuals with fewer sex partners (*B* = −0.079, *p*<0.05). Johns who had had STD infections showed a marginally significant level of more condom use (*B* = 0.504, *p*<0.1). However, in FSWs, condom use was more prevalent in individuals of a younger age (*B* = −0.009, *p*<0.05), undertaking non-inebriated sex (*B* = −0.153, *p*<0.001), with more sex partners (*B* = 0.005, *p*<0.01), with greater anxiety over STDs (*B* = 0.100, *p*<0.001), with higher self-efficacy (*B* = 0.410, *p*<0.001), and with no experience of STD infections (*B* = −0.133, *p*<0.01). Condom use tended to be higher, albeit at a marginally significant level, in the event of stronger social support (*B* = 0.002, *p*<0.1) and less smoking (*B* = −0.041, *p*<0.1). FSWs thus tended to seek safe sex for self-protection as they are obliged to have many sex partners due to their frequent sexual relationships for occupational reasons.

### Path Analyses of Influential Factors on Condom Use

**Figure 2:**
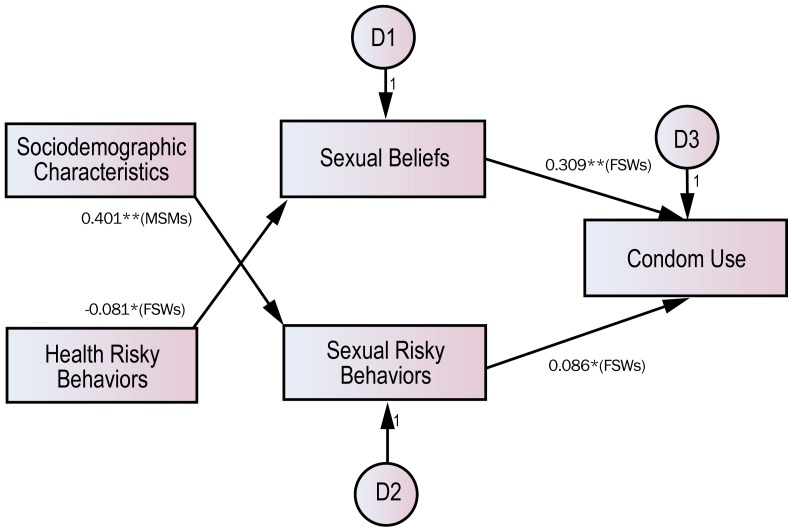
Results of path analyses of influential factors on condom use among those who have never had STDs. Note: The model does not presume any correlation among independent variables and displays significant paths only. *p<0.01; **p<0.001.

Path analyses were conducted to examine simultaneously the relationship among social factors and the mediating effect in CUBs caused by the experience of STDs. As seen in Figure 2, those who had not had STDs displayed a high level of sexual risk behavior (*B* = 0.086, *p*<0.05) and strong sexual belief (*B* = 0.309, *p*<0.01) as evidenced by FSWs, and the probability of condom use was higher in the case of non-execution of health risk behavior (*B* = **−**0.081, *p*<0.05). In FSWs, however, sexual risk showed the opposite direction of coefficient compared with health risk behavior in that it is reasonable to construe sexual risk as a matter of situation rather than of individual choice.

**Figure 3:**
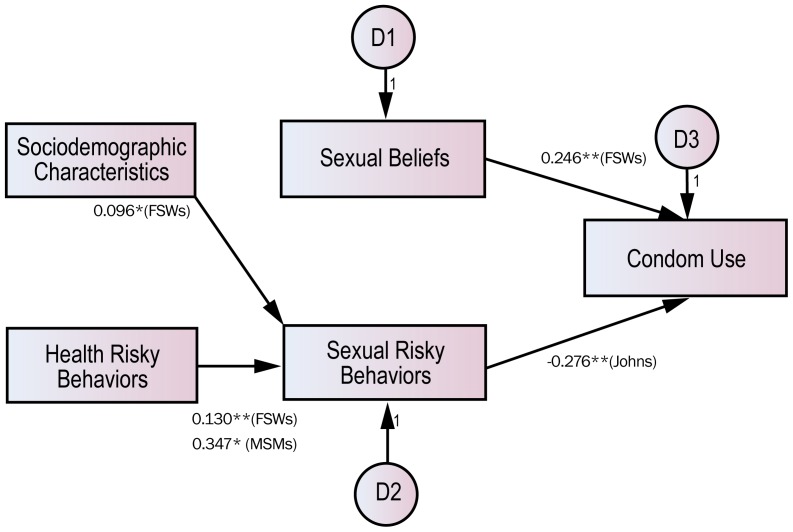
Results of path analyses of influential factors on condom use among those who have had STDs. Note: The model does not presume any correlation among independent variables and displays significant paths only. *p<0.01; **p<0.001.

Such patterns can vary considerably among those who had had STDs (Figure 3). FSWs had a higher probability of condom use only when sexual belief remained strong (*B* = 0.246, *p*<0.01). In other words, the experience of STDs affected condom use, and strong sexual belief acted as a factor motivating the practice of safe sex after infection. In Johns, however, the probability of condom use increased with less sexual risk behavior (*B* = **−**0.276, *p*<0.05). Therefore, the experience of STDs contributed to reducing sexual risk behavior but boosting condom use.

## Discussion

The present study attempted to explain the CUB of high-risk sexual groups that are vulnerable to STDs by focusing not on the psychological mechanisms, as is the case in the TPB and HBM theories, but on sexual risk behaviors. Of course, to overcome the low correlation between attitude and behavior, existing theories likewise argue that individuals possess subjective norms that reflect the attitudes of their significant others [30]. Such a model clearly predicts the CUB of general population groups, including immigrants [31], [32]. However, this model is relatively deficient in consideration of the specific contexts of sexual relations such as sexuality and the degree of the pursuit of sexual risk behavior. In particular, in the case of relations with casual partners instead of regular partners, the agents have sexual relations in the sex market, which has its own inherent logic and system [33], and therefore engage in condom negotiations or conform to the rules within the system [22], [34], [35]. As for groups with high sexual risks, CUBs cannot be properly understood without considering such contexts.

According to the present study, condom use was higher for MSMs when they were bisexuals and had high self-efficacy, for Johns when they had experience of STD infection, and for FSWs when they had high self-efficacy, when they did not engage in inebriated sex, and when they were anxious about contracting an infection. Regardless of whether or not they were infected with STDs, FSWs always used condoms when they had high sexual beliefs. In contrast, Johns exhibited a negative relationship between sexual risk behavior and condom use when they had experience of STD infection. The variable commonly significant to all three groups was the number of sex partners; but it exhibited a positive relationship with MSMs and Johns, and a negative relationship with FSWs.

The following social contexts in which each group practiced safe sex were reconstructed based on the study findings. MSMs were likely to use condoms when they were of a certain age, had considerable self-efficacy, or had few sex partners but had both same-sex and opposite-sex sexual relations. For them, however, there was almost no relationship between STD infection experience and CUB, and their respective sexual identities seemed to play an important role in condom use. The condom use of Johns was not explained by any particular predictors but was considerably affected by STD infection experience. Although Johns engaged in CSPs more often than did the general population groups, they reduced CSPs and increased condom use when they had experience of STD infection. Unlike the other two groups, who actively pursued sexual pleasure, FSWs exhibited a tendency to protect themselves from STDs through condom use. Consequently, whereas MSMs and Johns exhibited a risk-taking tendency in not using condoms as their number of sex partners increased, FSWs were stricter about their use of condoms as their number of sex partners increased. However, the experience of STD infection weakened the level of safe sex practices even for FSWs. This is because FSWs with STD infection experience had spent a relatively longer time in RLDs/brothels and had a gradual decrease in self-efficacy.

When sexual behavior was risky, the probability of STD infection increased significantly. Although it is well known that condom use helps prevent STDs, condom use in the present study was related to the riskiness of sexual behavior, and the experience of STD infection mediated the relationship between the two. Since the absence or presence of past STD experience was correlated with future STD infection probability, these two causal circles present new perspectives for understanding the sexual behavior of high-risk groups.

The present study had some limitations. First, the samples may not be representative of the classes of individuals being considered. In particular, a small sample of Johns gathered from a John School is unlikely to be representative. At the same time, there would be an inherent bias in collecting the sample. However, the number of sex partners and condom use rates of the sample were generally similar to the figures reported in earlier South Korean studies [3], [36]. Third, while the present study has proposed a condom use model based on theory, causal directionality and correlation need to be confirmed with more empirical evidence. Lastly, we need to consider reporting bias on the measurement of condom use, because it is likely to differ by many of the socio-demographic factors used to characterize the study groups.

### Conclusions

The findings of this study revealed that CUBs were related to sexual beliefs as well as sexual risk behaviors. At the same time, the experience of STD infection mediated the relationship between the two. Diverse implications may flow from the fact that condom use in high-risk population groups is a contextual decision that reflects individuals’ beliefs and unique sexual behaviors rather than being a planned or reasoned behavior. Even though condoms are tools for preventing STDs, the fact that condom use differs according to the presence or absence of infection with STDs is expected to facilitate more future studies as well as safer sex [37]–[39]. The empirical findings of this study not only help to understand groups vulnerable to sexual disease, but also help to suggest implications for public health in terms of effective methods for the prevention of sexual disease. To protect these minorities from STDs, we need to draw social attention towards promoting safer sex among groups vulnerable to STD infection.
